# Murine Models of B-Cell Lymphomas: Promising Tools for Designing Cancer Therapies

**DOI:** 10.1155/2012/701704

**Published:** 2012-02-12

**Authors:** Sabrina Donnou, Claire Galand, Valérie Touitou, Catherine Sautès-Fridman, Zsuzsanna Fabry, Sylvain Fisson

**Affiliations:** ^1^Institut National de la Santé et de la Recherche Médicale (INSERM), UMRS 872, Équipe Microenvironnement Immunitaire des Tumeurs, Centre de Recherche des Cordeliers, 75006 Paris, France; ^2^Université Pierre et Marie Curie-Paris 6, UMRS 872, 75006 Paris, France; ^3^Université Paris Descartes, UMRS 872, 75006 Paris, France; ^4^Service d'Ophtalmologie, Hôpital de la Pitié-Salpêtrière, AP-HP, 75013 Paris, France; ^5^Department of Pathology, University of Wisconsin, School of Medicine and Public Health, Madison, WI 53706, USA; ^6^Généthon, Evry, France; ^7^INSERM, UMRS 951, Evry, France; ^8^University of Evry Val d'Essonne, UMRS 951, Evry, France

## Abstract

Human B-cell lymphomas, the fourth most common hematologic malignancy, are currently the subject of extensive research. The limited accessibility of biopsies, the heterogeneity among patients, and the subtypes of lymphomas have necessitated the development of animal models to decipher immune escape mechanisms and design new therapies. Here, we summarize the cell lines and murine models used to study lymphomagenesis, the lymphoma microenvironment, and the efficacy of new therapies. These data allow us to understand the role of the immune system in the fight against tumors. Exploring the advantages and limitations of immunocompetent versus immunodeficient models improves our understanding of the molecular and cellular mechanisms of tumor genesis and development as well as the fundamental processes governing the interaction of tumors and their host tissues. We posit that these basic preclinical investigations will open up new and promising approaches to designing better therapies.

## 1. Introduction

**Figure 1:**
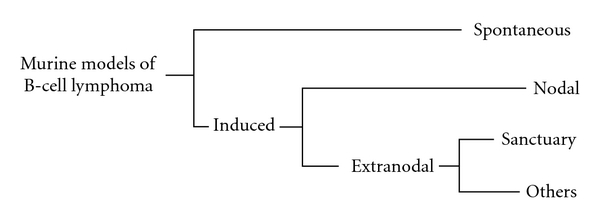
Schematic representation of the major subtypes of B-cell lymphoma murine models. Murine models can be either spontaneous and develop in genetically engineered mice or can be induced by implantation of a tumor cell line. In this case, it is possible to inject cells in lymph nodes (nodal location) or outside of them (extranodal location). Immune sanctuaries such as the brain or the eyes provide information about that particular situation, but many other sites can be envisaged.

Lymphomas are highly heterogeneous diseases, varying by both the type of malignant cell and the tumor location. They most frequently originate from B cells, and the two main groups of B-cell lymphomas, B-cell non-Hodgkin lymphomas (NHL) and Hodgkin lymphomas, account, respectively, for about 80% and 15% of all lymphomas. Of the NHL, half are diffuse large B-cell lymphomas, followed in prevalence by follicular lymphomas, marginal zone lymphomas, Burkitt's lymphomas, and mediastinal lymphomas. This heterogeneity makes it difficult to collect human samples in sufficient quantities for statistical analyses. Moreover, these samples are not easy to classify in the absence of clear discriminative parameters. In addition, some tumors, such as primary central nervous system (CNS) lymphomas, are located deep within delicate tissues, which complicates the collection of biopsy samples and complete tumor analysis [1]. Studying these tumors is thus quite challenging. Animal models are very useful, because they let us work on very homogeneous materials. They are also essential for preclinical studies and allow us to perform kinetic analyses together with detailed investigation of the tumors' characteristics and microenvironments. Here, we will review the spontaneous and induced B-cell lymphoma models that can occur in transgenic mice, or by various types of transfer of tumor cells into wild-type mice (Figure 1). We will summarize the known categories of B-cell lymphoma mouse models and discuss their experimental and translational values. Finally, we will examine how the tumors regulate their microenvironment in different tissues and how this knowledge could be translated into practical applications for tumor therapies.

## 2. Models for Studying Lymphomagenesis

One of the key questions about tumor development concerns the origin and the mechanisms responsible for malignant phenotypes. Various spontaneous tumor models have been developed to study how B-cell lymphomas arise and mature in different tumor environments. Observations and experiments with human tissue samples have provided some indications about the possible genetic events that might be responsible for uncontrolled B-cell proliferation. Recent advances in genetic engineering have made it possible to develop transgenic mouse models recapitulating major known modifications of the genome and to infect mice with viruses that can induce B-cell lymphomas.

The myc oncogene is the gene most frequently studied: its translocation behind an enhancer or promoter region specific for B lymphocytes can give rise to B-cell lymphomas (Table 1). The involvement of such a translocation in lymphomagenesis is studied in the most used mouse model, E*μ*-Myc. In this transgenic experimental tumor model, when the myc gene is inserted into the IgH locus, B-cell lymphomas develop at a 100% incidence rate. Nonetheless, disease onset is, as in humans, highly variable (from day 32 to day 600), as is the phenotype of the tumors in different mice. More precisely, Mori and colleagues [14] have described two principal tumor phenotypes: the first type arises during an early time window and is composed mainly of immature B cells, thus resembling Burkitt's lymphoma. The second type develops very late (after day 400) and is composed of mature B cells; it is similar to diffuse large B-cell lymphomas [14]. Interestingly, if the myc gene is placed under the enhancer region of the Ig light chain genes, it results in a disease very similar to Burkitt's lymphoma in humans [7] (Table 1). Sheppard and colleagues [4] generated another transgenic mouse with the translocation of the N-myc gene under the IgH enhancer and with only a subtle modification of the endogenous myc expression level. This resulted in an indolent disease and only 25% incidence after 9 to 12 months. After infection with the murine Moloney leukemia virus, however, both the incidence and speed of tumor development were far greater. Following this idea, others developed a model that uses the Brd2 gene and can induce B-cell lymphoma in some mice after translocation but is not sufficient to obtain a high incidence. Modification of these mice by infection with a retrovirus expressing the ras oncogene also aggravated and accelerated lymphomagenesis [10] (Table 1). These results point out that translocation of the oncogene by itself is not sufficient to create a malignant phenotype. The added value of these transgenic models is that tumors develop on a syngeneic background that makes it possible to analyze the tumor microenvironment and its influence on tumor growth. For example, by studying the influence of Bcl2 overexpression in the hematologic compartment in generating a follicular B-cell lymphoma, Egle and collaborators [8] were able to determine that CD4^+^ T cells were crucial in the proliferation of germinal center B cells and therefore in lymphomagenesis.

To understand the mechanisms linked to lymphomagenesis in more detail, researchers have introduced more strategies to transfer variously modified tumor cells into immunodeficient or immunocompromised hosts (models listed in Tables 2 and 3). These approaches introduce the potential bias of tumor injection to specific tissue sites and are unable to follow the progressive induction and development of tumors from a few malignant cells. Despite these weaknesses, tumor injection models are very useful because they let us study the impact of different mutations on tumor aggressiveness (models listed in Table 2). For example, the potential role of pax5 in lymphomagenesis was studied with cell lines deficient for this gene [17]. Using the same idea, Yu and collaborators [18] developed a strategy to determine the influence of different genes in enhancing the tumor-inducing potential of the myc gene translocation (Table 3). By mixing bone marrow from p53 null mice with a packaging cell line producing the myc-encoding retrovirus, they demonstrated that p53 inactivation together with myc overexpression was sufficient to induce B-cell lymphomas. These models could easily and rapidly be adapted to help define the effect of other genes and gene interactions on lymphomagenesis without the need to develop transgenic mice. 

The diversity of the animal models listed in Tables 1–3 makes it very challenging to study the influence of different characteristics on the development of specific types of tumors in experimental animals and to draw significant conclusions about human B-cell lymphoma development. Some models are heterogeneous [14] or can only be classified depending on the differentiation stage of the tumor cells used [47], while some transgenic mice are clearly associated with specific B-cell lymphoma phenotypes. Some models are nonetheless very useful for studying human disease. For example, because NFS.V mice develop tumors that are very similar to marginal zone lymphomas [5], they provided a basis for defining its stages of progression (Table 1). Another highly relevant tumor model is the blastoid variant of mantle cell lymphoma, developed by Ford and colleagues [12] by generation of double-transgenic mice for the IL-14a and myc genes (Table 3). A more recent model with the full genome of the hepatitis C virus introduced into CD19-expressing cells spontaneously develops human diffuse large B-cell lymphoma [15]. A nontransgenic strategy also led to the development of a model of mucosa-associated lymphoid tissue lymphoma, shown to recapitulate most human disease characteristics [48]. 

The major advantage of the after mentioned models is that they develop through multiple spontaneous genetic events that will help us to discover novel mechanisms of tumorigenesis. At the same time, they are also associated with some experimental limitations. First, time to onset of disease varies enormously within and between models (from day 32 to day 600 for some models), precluding the assessment of new therapies in these conditions. Moreover, as described previously, tumor incidence is high but disparate, up to 100% in some cases, but not higher than 25% in others. Furthermore, in a given model system, these tumors can vary greatly in tumor location and phenotype, which makes it difficult to compare different animals in the same experiment. While it is clear that spontaneous B-cell lymphoma models provide unique insight into tumor development, we must nonetheless bear in mind that the *in vivo* tumor generation process is quite complex. These models, together with the adoptive tumor transfer models, will be critical to our understanding of lymphomagenesis.

## 3. Models to Study the Lymphoma Microenvironment

The tumor microenvironment is an essential and complicating aspect of a tumor that must be better understood if more targeted treatments are to be developed [49]. Studying all the features of a malignancy requires working on syngeneic models. Moreover, reproducible models with well-characterized tumor development are important for the analysis of immune response, which remains impossible with spontaneous models. Two main questions must be addressed in developing such models: the tumor cells to be injected and the site of tumor injection, that is, nodal or extranodal, in the peripheral or central nervous system. The tumor cells are of human origin and therefore implanted into immunodeficient mice, or, more often, they are syngeneic to their host, so the tumor-induced immunity can be studied (Table 2). Lymphomas can invade many different organs in humans, especially secondary lymphoid organs and the central nervous system. Tumor cells may be injected into these organs in the mouse or directly into the blood; the latter allows spontaneous tumor colonization to different locations. The advantages of targeting a specific tissue include the possibility of comparing tumor growth between different microenvironments and determining the relative roles in tumor development of the tissue characteristics and the intrinsic tumor cell characteristics [50].

**Figure 2:**
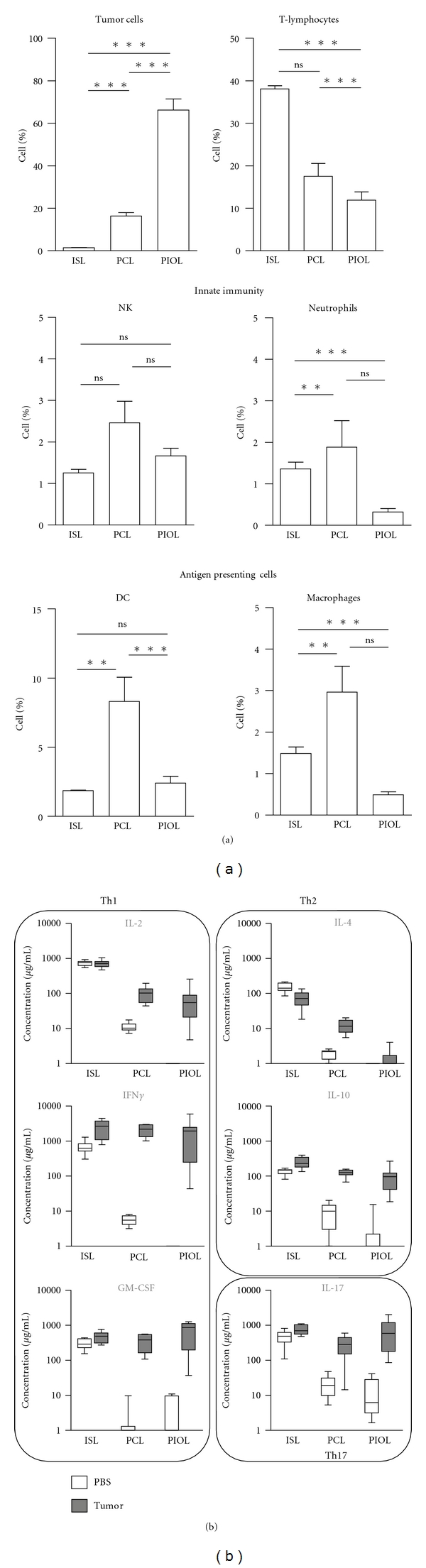
Comparison of the cellular and molecular immune environment of a B cell murine lymphoma implanted in the spleen, in the brain or in the eye. (a) A20.IIA-GFP cells were implanted in immunocompetent syngeneic mice in the spleen (intrasplenic lymphoma model: ISL), in the brain (primary intracerebral lymphoma model: PCL), or in the eye (primary intraocular lymphoma model: PIOL). 21 days after injection, tumor-bearing organs were analyzed by flow cytometry for the presence of GFP^+^ tumor cells, CD3^+^ T lymphocytes, NKp46^+^ NK cells, Gr1^+^ neutrophils, CD11c^+^ dendritic cells, and CD11b^+^CD11c^−^ macrophages. Results are represented as the proportion of the different populations among total living cells (*n* = 10). (b) 21 days after lymphoma (gray boxes) or PBS (white boxes) injection, cells were isolated from appropriate tissues and stimulated for 36 h with anti-CD3/CD28-coated Dynal beads. Secretion of IL-2, IFN*γ*, GM-CSF, IL-4, IL-10, and IL-17 in the culture supernatant was evaluated by cytokine bead arrays (BD Biosciences) (*n* = 10). Animal studies were conformed to European Union guidelines and were approved by the Charles Darwin Ethics Committee in Animal Experiment, Paris, France.

To study the microenvironment of B-cell lymphomas in different tissues, we implanted a tumor cell line derived from the well-known A20 tumor into different tissue locations, including the spleen, brain, and eyes, in syngeneic mice [45, 50]. As Figure 2 illustrates, T cells infiltrated the tumor at each location, even immune-privileged tissues, and represented up to 15% of all live cells in these sites. Moreover, antigen-presenting cells also infiltrated into the A20.IIA-GFP tumor, particularly in the brain where CD11c^+^ dendritic cells and CD11b^+^CD11c^−^ macrophages accounted for a higher proportion of cells than in the tumor-bearing eye or spleen. Innate immune cells were also found in the tumor microenvironment, especially in the brain and spleen (Figure 2). The absence of spontaneous tumor rejection in these experiments indicates an immunosuppressive environment. Strikingly, nonetheless, even immune-privileged sites such as the eye or the brain were able to induce an immune response, with cellular and molecular environments similar to those of peripheral tissue sites. These findings suggest that the primary regulator of the tumor microenvironment is the tumor itself rather than the local tissue structure.

Even so, we note some features specific to CNS tumors, in particular the delay in the infiltration of T cells, especially CD8^+^ T cells, into the eye and the brain. Moreover, we observed among these infiltrated T cells a large proportion of CD4^+^CD25^+^Foxp3^+^ regulatory T cells, accounting for up to 40% of all CD4^+^ T lymphocytes, compared with no more than 20% in the spleen [50]. In related findings, Elpek and colleagues [74], using the parental A20 cell line implanted subcutaneously, highlighted the importance of regulatory T cells in the early phase of tumor growth. Curti's group [60] used the same cell line in an intrasplenic tumor model to study the accumulation of regulatory T cells in the spleen and showed that the IDO enzyme is critical for the local conversion of conventional T cells into regulatory ones. Serafini's team [75] showed that a robust expansion of specific regulatory T cells follows intravenous injection of A20 cells and demonstrated that a population of myeloid-derived suppressive cells is responsible for this expansion. Others have also suggested that myeloid-derived cells can contribute to the tumor immune response, as when M2 macrophages infiltrate the B-cell lymphoma growing in the brain after implantation of human Raji cells into nude mice [76].

All these results show that B-cell lymphomas induce immunosuppressive cells, although how these cells contribute to tumor growth remains unknown. It is assumed that suppressive myeloid cells can influence the molecular milieu of the tumor. We and others have shown the production of various anti-inflammatory mediators, depending on the experimental setting, including IL-10 [74], IL-4 [76], and soluble receptor for IL-2 [15]. Additionally, as Figure 2 shows, we found that T cells from tumor-bearing mice do not produce the proinflammatory cytokines IFN*γ* or GM-CSF without stimulation, except in the spleen where resident T cells might be responsible for this secretion. After polyclonal stimulation we observed an unbalanced Th1/Th17 profile, with high levels of IFN*γ*, GM-CSF, and IL-17 and low levels of IL-2, IL-4, and IL-10 [45, 50].

In summary, it is clear that tumor cells closely regulate the microenvironment of B-cell lymphomas. It is advantageous for the tumor to generate a suppressive environment for optimal tumor growth. Understanding the balance between pro- and anti-inflammatory mediators that can contribute to or control tumor growth is essential for designing novel tumor therapies.

## 4. Models to Analyze the Efficacy of New Therapies

### 4.1. Assessment of Treatment Efficacy

In recent years, tumor therapies have achieved substantial but still incomplete success. It is generally accepted that well-characterized human tumor cell lines and *in vivo* animal models are required to develop novel antitumor treatments. Basically, four types of models have been developed (Table 3): (i) syngeneic murine models, (ii) syngeneic models with murine tumor cells engineered to express human antigens, (iii) human cells implanted into immunodeficient mice, and (iv) humanized mice, that is immunodeficient mice reconstituted with human immune system and then implanted with human tumor cells.

The standard treatment for aggressive B-cell malignancies is the combination of four chemotherapy agents (i.e., cyclophosphamide, doxorubicin, vincristine, and prednisolone) with rituximab, also called R-CHOP therapy [77]. Because B-lymphoma cells express the CD20 antigen, they are a suitable target for anti-CD20 monoclonal antibodies (mAb) such as rituximab. The use of this chimeric mAb has enhanced the survival of patients with different B-cell malignancies, after various studies confirmed its therapeutic potential. Most of those studies used murine models of human tumor cell lines implanted into immunodeficient mice. For example, the efficacy of rituximab against disseminated Burkitt lymphoma Daudi cells and against DLBCL SU-DHL4 cells was assessed in SCID mice, and the therapeutic advantages varied with the cell line [59]. Hernandez-Ilizaliturri and colleagues [78] also used SCID mice, with the Raji cell line, and obtained better results: rituximab treatment enabled 60% of the animals to reject their tumors. This great potential has led many laboratories to seek to improve this efficacy by designing new engineered antibodies, such as EMAB-6 [79] or the humanized GA101 used against the human SU-DHL4 tumor implanted into SCID/beige mice [80]. Coupling rituximab with other therapies has also been evaluated in these murine models of human tumors, as, for example, with R-CHOP therapy [81], or the TLR9 agonist CpG against Daudi cells [82]. Advances in understanding the biology of tumor cells and the tumor microenvironment have led to the design of new therapies, combined or not with preexisting ones (for review, see [83]). All of these strategies have been evaluated by implanting human cell lines into immunodeficient mice. They include the application of immunomodulatory drugs ([84]: subcutaneous Raji cells into SCID mice), inhibition of antiapoptotic signals ([65]: different cell lines into SCID or SCID/beige mice), or inhibition of specific metabolic or signaling pathways ([69]: intracerebral Raji cells into nude mice; [46]: subcutaneous HKBML cells into SCID mice). New strategies are also evaluated in this way, including the use of immunoconjugates ([73]: intraocular CA46 cells into SCID mice; [85]: intravenous Raji cells into SCID mice), or reoviruses, which target cells expressing high levels of the ras oncogene ([86]: subcutaneous Daudi and Raji cells into SCID/nod mice).

The major disadvantage of the experimental models described in this subsection is that they involve immunodeficient hosts that lack the adaptive immunity present in tumor microenvironment in humans, that do not reflect the complexity of human diseases.

### 4.2. Assessment of Immune System Involvement in Tumor Rejection

The role of the immune system is essential in tumor rejection. Syngeneic models, besides their utility in analysis of the tumor microenvironment, are particularly helpful for studying how specific treatments modulate the immune system or particular components of it. One of the indicators studied most frequently after treatment is the quantity of T lymphocytes, especially CD8^+^ T cells, infiltrating the tumor [87]. In an A20 B-cell lymphoma model implanted subcutaneously, treatment by survivin or an idiotype-binding peptide, is correlated with increased CD8^+^ T-cell infiltration [63, 88]. Some therapies are clearly designed to boost immune response. For example, in the 4TOO intravenous model, administration of a vaccine composed of a fusion between dendritic cells and tumor cells increases T-lymphocyte proliferation and promotes the secretion of IL-2, IL-6, and IFN*γ* (model listed in Table 3). In this model, significant amounts of IL-17 are found, which suggests that Th17-expressing cells contribute to tumor rejection [55]. Houot and Levy [87], in a two-site subcutaneous A20 model, tried to design an antitumor therapy based on the intratumoral inoculation of CpG and two T-cell-modulating mAbs. They obtained strong response rates and were able to demonstrate the involvement of CD4^+^ T cells in controlling the primary tumor site and the role of CD8^+^ T cells in controlling distant tumors.

Another significant advantage of murine models is the abundance of different mutant mice, deficient for well-characterized molecules or cell populations. For example, a subcutaneous Raji lymphoma model implanted into Fc*γ*-receptor-deficient mice demonstrated the importance of Fc receptors in the therapeutic efficacy of cytotoxic antibodies and the abolition of the anti-CD20 rituximab antitumor effect [89]. Additionally, Flynn and Stockinger [90] studied the role of specific CD4^+^ populations in the subcutaneous LK35 tumor model with Rag^−/−^ *γ*c^−/−^ mice that lack all lymphocytes and NK cells. They demonstrated that memory T cells were capable of controlling tumor growth initially, without the help of other components of the immune system. However, immune pressure eventually led to selection of tumor cells unable to present antigens, which resulted in turn in paralysis of the T-cell response.

Another potential way to obtain data more relevant to human tumors is to use murine cell lines expressing specific human tumor antigens, which would make it possible to test monoclonal antibodies against specific antigens. For example, the murine 38C13 cell line expressing the human CD20 antigen has been used to evaluate the therapeutic potential of rituximab against intravenously injected disseminated tumors [43] and against central nervous system tumors [68]. The authors showed in the first case [43] that depletion of neutrophils, NK cells, and macrophages did not influence antibody efficacy, but complement inhibition abolished this effect. More recently, others have used the EL4 thymoma cell line transfected with this same human CD20 antigen and the luciferase gene to monitor tumor regression after rituximab therapy [91, 92]. For now, the best system for studying tumor rejection appears to involve the reconstitution of immunodeficient mice with human immune cells to generate humanized mice, followed by the implantation of human tumor cells into these experimental animals. Sato and collaborators [93] developed this model to define the role of the complement system in the efficacy of an optimized variant of rituximab. In another example, human Daudi cells were implanted into SCID mice that were reconstituted with human peripheral blood leukocytes to assess the efficacy of a vaccine composed of immature dendritic cells and antihuman CD40 mAb [94]. This study demonstrated the rate at which activated CD8^+^ cytotoxic T lymphocytes infiltrate Burkitt lymphomas and the high level at which these cells secrete IFN*γ* after the injection of this vaccine. More recently, a model was developed to test the efficacy of an agonistic anti-CD40 antibody against different subcutaneous B-cell tumor cell lines, such as Daudi, Raji, and Jijoye, implanted into SCID/beige mice. Although this type of treatment by itself reduced tumor size, it was much more effective after reconstitution of the mice with human T cells and dendritic cells [66].

In summary, antitumor immune responses hold great promise for boosting tumor therapy. More appropriate models are needed to explore the possibility of dendritic cell vaccination therapies in combination with specific tumor cell targeting.

## 5. Conclusion

Recent experimental animal models that allow us to study the induction and development of human tumors are important achievements. Only with a deeper understanding of the molecular and cellular mechanisms leading to tumor genesis and development of tumor microenvironments can we design better therapies. Despite the advances with the animal models described in this paper, several questions remain open. Given the difficulty in comparing murine tumors and human malignancies, more relevant models are needed. As this paper shows, there are many different models that seek to mimic human disease, but no consensus exists for any given model. Most tumor cells and spontaneous models require better characterization from histologic, phenotypic, genetic, and immunologic perspectives. Parallels with human diseases are also complicated by the absence of a clear classification of B-cell lymphomas. One interesting future challenge will be to develop a humanized murine model that can be implanted with human tumors and reconstituted with a complete human immune system for each main subclass of B-cell lymphomas. The remarkable impact that these models have had on the development of novel tumor therapies justifies the aggressive pursuit of basic and preclinical investigations to develop more appropriate animal models and unravel the fundamental processes governing the interaction of tumors with host tissues.

## Figures and Tables

**Table 1:** Spontaneous models of B-cell lymphoma. B-NHL: non-Hodgkin B-cell lymphoma; CLL: chronic lymphocytic leukemia; DLBCL: diffuse large B-cell lymphoma; DTG: double transgenic mice; n.d.: not determined.

Name	Lymphoma subtype/orgin	Strain (haplotype)	Major reference
B10 H-2^a^ H-4^b^p/Wts	CLL	C57Bl/10 (H-2^b^)	[2]
SL/KH	Pre-B lymphoma	SL/KH (H-2^11^)	[3]
E*μ*-N-myc	Indolent B-NHL	C57Bl/6 x DBA/2 (H-2^b/d^)	[4]
NFS.V+	Marginal zone lymphoma	NFS.V+ (H-2^sq4^)	[5]
NMRI/RFB-MuLV	n.d.	NMRI (H-2^q^)	[6]
B6-l-MYC	Burkitt-like lymphoma	C57Bl/6 (H-2^b^)	[7]
VavP-Bcl2	Follicular lymphoma	C57Bl/6 (H-2^b^)	[8]
Lig4/p53 KO	Pro/Pre-B lymphoma	C57Bl/6 × sv129	[9]
E*μ*-BRD2	DLBCL	FVB (H-2^q^)	[10]
Bcl6 Knock in	Germinal center, DLBCL	C57Bl/6 × sv129	[11]
Bcl6/Myc transgenic	Post germinal center, DLBCL	C57Bl/6 × sv129	[11]
IL-14aTGxc-Myc TG (DTG)	Blastoid variant of mantle-cell lymphoma	C57Bl/6 (H-2^b^)	[12]
Myc/BCR^HEL^/HEL	Burkitt-like lymphoma	C57Bl/6 (H-2^b^)	[13]
E*μ*-myc	From follicular to DLBCL (time dependant)	C57Bl/6 × sv129	[14]
RzCD19Cre	NHL, hepatitis C induced	129/Sv (H-2^bc^); BALB/c (H-2^d^); C57Bl/6 (H-2^b^)	[15]
UVB induced	Mature B-cell lymphoma	C57Bl/6 p53^+/−^ (H-2^b^)	[16]

**Table 2:** Most common B lymphoma cell lines. B-NHL: non-Hodgkin B-cell lymphoma; DLBCL: diffuse large B-cell lymphoma; N/A: not attributable.

Name	Orgin	Strain (haplotype)	Reference
L1210	Ascitic fluid of 8 months mouse, lymphocytic leukemia cells	DBA/2 (H-2^d^)	[19]
Raji	Burkitt lymphoma from an 11-year-old child (maxilla)	N/A	[20]
Jijoye	Lymphoblastic cell line derived from a 7-year old boy with Burkitt lymphoma EBV^+^	N/A	[21]
Daudi	16-year-old black male with Burkitt lymphoma, orbital tumor	N/A	[22]
Ramos	Burkitt lymphoma	N/A	[23]
BJAB	Burkitt lymphoma	N/A	[24]
SU-DHL-4	DLBCL patient (peritoneal effusion of woman with B-NHL)	N/A	[25]
38C13	Carcinogen induced	C3H/HeN (H-2^k^)	[26]
BCL1	Spontaneous	BALB/c (H-2^d^)	[27]
A20	Spontaneous reticulum cell sarcoma of an old mouse	BALB/c (H-2^d^)	[28]
CA46	Ascite fluid of a patient with Burkitt lymphoma	N/A	[29]
MC116	Undifferenciated B cell lymphoma	N/A	[29]
4TOO	Plasmacytoma originating from MPC-11 cells	BALB/c (H-2^d^)	[30]
B6 spontaneous model	Spontaneous	C57Bl/6 (H-2^b^)	[31]
L3055	Burkitt's lymphoma of germinal center origin	N/A	[32]
SC-1	Burkitt lymphoma	N/A	[33]
CH44	Follicular center cell lymphoma derived from B10.H-2a/H-4bp/Wts, large cell type	B10.H-2^a^H-4^b^p/Wts (H-2^a^-4^b^)	[34]
DoHH-2	Pleural fluid of 60-year old man with centroblastic non-Hodgkin lymphoma	N/A	[35]
S11	From Gammaherpesvirus-68-infected mice	BALB/c (H-2^d^)	[36]
LY-ar / LY-as	Derived from the spontaneous LY-TH tumor	C3Hf/kam	[37]
Granta 519	Peripheral blood at relapse of high grade B-NHL	N/A	[38]
Pi-BCL1	Prolymphocytic, foetal liver derived	BALB/c (H-2^d^)	[39]
38C13 Her2/neu	Carcinogen induced	C3H/HeN (H-2^k^)	[40]
Myc5-M5	Derived from a tumor induced in p53 null mice infected with myc encoding retrovirus	C57Bl/6 (H-2^b^)	[18]
Mouse lymphosarcoma cell line	Nitrosomethylurea induced	CBA (H-2^k^)	[41]
FL5.12 transfected by Bcl2	IL-3-dependant BALB/c pro-B cell line	BALB/c (H-2^d^)	[42]
38C13 CD20^+^	Carcinogen induced	C3H/HeN (H-2^k^)	[43]
Z138	Mantle cell lymphoma with blastoid transformation	N/A	[44]
A20.IIA-GFP / IIA1.6-GFP	Reticulum cell sarcoma	BALB/c (H-2^d^)	[45]
HKBML	Brain lymphoma	N/A	[46]

**Table 3:** Induced models of B-cell lymphoma. (m): murine origin; (h): human origin; (i): syngeneic models; (ii): syngeneic models with murine tumor cells engineered to express human antigens; (iii): xenogenic models; (iv): humanized models; CLL: chronic lymphocytic leukemia; DLBCL: diffuse large B-cell lymphoma; MALT: mucosa associated lymphatic tissue; n.d.: not determined; PCL: primary cerebral lymphoma; PCNSL: primary central nervous system lymphoma; PIOL: primary intraocular lymphoma; SCID: severe combined immune deficiency.

Injection site	Name	Lymphoma model	Recipient mice			Major reference
			Strain (haplotype)	MHC compatibility	Immune status	
Intravenous	B6 spontaneous model (m)	High-grade B lymphoma	C57Bl/6 (H-2^b^)	Syngeneic (i)	Immunocompetent	[51]
	Pi-BCL1 (m)	DLBCL	BALB/c (H-2^d^)	Syngeneic (i)	Immunocompetent	[52]
	38C13 (m)	Non-Hodgkin lymphoma	C3H/HeN (H-2^k^)	Syngeneic (i)	Immunocompetent	[53]
	FL5.12 transfected by Bcl2 (m)	Non-Hodgkin lymphoma	BALB/c (H-2^d^)	Syngeneic (i)	Immunocompetent	[42]
	A20 (m)	DLBCL	BALB/c (H-2^d^)	Syngeneic (i)	Immunocompetent	[54]
	4TOO (m)	n.d.	BALB/c (H-2^d^)	Syngeneic (i)	Immunocompetent	[55]
	BCL1 (m)	CLL	BALB/c (H-2^d^)	Syngeneic (i)	Immunocompetent	[56]
	38C13 Her2/neu (m)	Non-Hodgkin lymphoma	C3H/HeN (H-2^k^)	Syngeneic (ii)	Immunocompetent	[40]
	Z138 (h)	Human mantle cell lymphoma	SCID mice (H-2^d^)	Xenogenic (iii)	Immunodeficient	[57]
	BJAB (h)	Burkitt lymphoma	SCID mice (H-2^d^)	Xenogenic (iii)	Immunodeficient	[58]
	SU-DHL-4 (h)	DLBCL	SCID mice (H-2^d^)	Xenogenic (iii)	Immunodeficient	[59]

Intrasplenic	A20 (m)	DLBCL	BALB/c (H-2^d^)	Syngeneic (i)	Immunocompetent	[60]
	A20.IIA-GFP (m)	DLBCL	BALB/c (H-2^d^)	Syngeneic (i)	Immunocompetent	[50]

Intraperitoneal	CH44 (m)	Non-Hodgkin lymphoma	B10.H-2^a^H-4^b^p/Wts	Syngeneic (i)	Immunocompetent	[34]
	BCL1 (m)	DLBCL	BALB/c (H-2^d^)	Syngeneic (i)	Immunocompetent	[53]
	38C13 (m)	Non-Hodgkin lymphoma	C3H/HeN (H-2^k^)	Syngeneic (i)	Immunocompetent	[53]
	Tonsillar lymphocytes and EBV infection (h)	Viro-induced lymphoma	BNXhum (humanized)	Allogenic (iv)	Immunocompetent	[61]

Subcutaneous	LY-ar or LY-as (m)	n.d.	C3Hf/kam (H-2^k^)	Syngeneic (i)	Immunocompetent	[37]
	S11 (m)	Burkitt lymphoma	BALB/c nude (H-2^d^)	Syngeneic (i)	T-cell deficiency	[62]
	LMycSN-p53null (m)	Non-Hodgkin lymphoma	C57Bl/6 (H-2^b^)	Syngeneic (i)	Immunocompetent	[18]
	A20 (m)	DLBCL	BALB/c (H-2^d^)	Syngeneic (i)	Immunocompetent	[63]
	38C13 Her2/neu (m)	Non-Hodgkin lymphoma	C3H/HeN (H-2^k^)	Syngeneic (ii)	Immunocompetent	[40]
	Myc5-M5 (m)	n.d.	SCID mice (H-2^d^)	Allogenic	Immunodeficient	[17]
	Splenic Hodgkin lymphoma cells (h)	Hodgkin disease	Nude mice (H-2^b^)	Xenogenic (iii)	T-cell deficiency	[64]
	Human hodgkin cell line (h)	Hodgkin disease	SCID mice (H-2^d^)	Xenogenic (iii)	Immunodeficient	[47]
	Ramos (h)	Burkitt lymphoma	SCID mice (H-2^d^)	Xenogenic (iii)	Immunodeficient	[58]
	BJAB (h)	Burkitt lymphoma	SCID mice (H-2^d^)	Xenogenic (iii)	Immunodeficient	[58]
	SC-1 (h)	Follicular lymphoma	SCID mice (H-2^d^)	Xenogenic (iii)	Immunodeficient	[58]
	DoHH-2 (h)	Follicular lymphoma	SCID mice (H-2^d^)	Xenogenic (iii)	Immunodeficient	[58]
	SuDHL-4 (h)	DLBCL	C.B-17 SCID mice (H-2^d^)	Xenogenic (iii)	Immunodeficient	[65]
	Granta 519 (h)	Mantle cell lymphoma	C.B-17 SCID mice (H-2^d^)	Xenogenic (iii)	Immunodeficient	[65]
	HKBML (h)	Brain DLBCL	C.B-17 SCID mice (H-2^d^)	Xenogenic (iii)	Immunodeficient	[46]
	Daudi (h)	Burkitt lymphoma	SCID/beige mice (H-2^d^)	Xenogenic (iv)	Partially rebuilt	[66]
	Jijoye (h)	Burkitt lymphoma	SCID/beige mice (H-2^d^)	Xenogenic (iv)	Partially rebuilt	[66]

Intramuscular	MSV-MuLV-M induced	Waldenstrom's macroglobulinemia	C57Bl/6 (H-2^b^)	Syngeneic	Immunocompetent	[67]
	Mouse lymphosarcoma cell line (m)	Non-Hodgkin lymphosarcoma	CBA (H-2^k^)	Syngeneic (i)	Immunocompetent	[41]

Stomach	Helicobacter felis	MALT lymphoma	BALB/c (H-2^d^)	Syngeneic (i)	Immunocompetent	[48]

Intracerebral	A20.IIA-GFP (m)	PCL (PCNSL)	BALB/c (H-2^d^)	Syngeneic (i)	Immunocompetent	[50]
	38C13 CD20^+^ (m)	PCL (PCNSL)	C3H/HeN (H-2^k^)	Syngeneic (ii)	Immunocompetent	[68]
	Raji (h)	PCL (PCNSL)	Nude mice (H-2^b^)	Xenogenic (iii)	T-cell deficiency	[69]
	Patient's cells (h)	PCL (PCNSL)	Nude mice (H-2^b^)	Xenogenic (iii)	T-cell deficiency	[70]
	MC116 (h)	PCL (PCNSL)	Nude rats (RT1^u^)	Xenogenic (iii)	Immunodeficient	[71]

Cisterna magna	L1210 (m)	Leptomeningeal metastases	DBA/2 (H-2^d^)	Syngeneic (i)	Immunocompetent	[72]

Intraocular	A20.IIA-GFP (m)	PIOL	BALB/c (H-2^d^)	Syngeneic (i)	Immunocompetent	[45]
	38C13 CD20^+^ (m)	PIOL	C3H/HeN (H-2^k^)	Syngeneic (ii)	Immunocompetent	[68]
	CA46 (h)	PIOL	SCID mice (H-2^d^)	Xenogenic (iii)	Immunodeficient	[73]

## Abbreviations

B-NHL: B-cell non-Hodgkin lymphomas
CLL: Chronic lymphocytic leukemia
CNS: Central nervous system
DLBCL: Diffuse large B cell lymphoma
MALT: Mucosa associated lymphatic tissue
PCL: Primary cerebral lymphoma
PCNSL: Primary central nervous system lymphoma
PIOL: Primary intraocular lymphoma
SCID: Severe combined immune deficiency.
